# Functional Severe Acute Respiratory Syndrome Coronavirus 2 Virus-Like Particles From Insect Cells

**DOI:** 10.3389/fmicb.2021.732998

**Published:** 2021-10-20

**Authors:** Antonina Naskalska, Agnieszka Dabrowska, Artur Szczepanski, Krzysztof P. Jasik, Beata Gromadzka, Krzysztof Pyrc

**Affiliations:** ^1^Virogenetics Laboratory of Virology, Malopolska Centre of Biotechnology, Jagiellonian University, Kraków, Poland; ^2^Department of Microbiology, Faculty of Biochemistry, Biophysics and Biotechnology, Jagiellonian University, Kraków, Poland; ^3^Department of Pathology, School of Pharmacy, Medical University of Silesia in Katowice, Sosnowiec, Poland; ^4^Department of “in vitro” Studies, Institute of Biotechnology and Molecular Medicine, Gdańsk, Poland; ^5^NanoExpo^®^, Gdańsk, Poland

**Keywords:** SARS CoV-2, virus-like particles, purification, transduction, insect cells

## Abstract

Severe acute respiratory syndrome coronavirus 2 (SARS-CoV-2) remains a major epidemic threat since the beginning of 2020. Efforts to combat the virus and the associated coronavirus disease 2019 (COVID-19) disease are being undertaken worldwide. To facilitate the research on the virus itself, a number of surrogate systems have been developed. Here, we report the efficient production of SARS-CoV-2 virus-like particles (VLPs) in insect cells. Contrary to widely used pseudovirus particles, where only one coronaviral protein is displayed within a heterologous scaffold, developed VLPs are structurally similar to the native virus and allow for more throughput studies on the biology of the infection. On the other hand, being devoid of the viral genome, VLPs are unable to replicate and thus safe to work with. Importantly, this is the first report showing that SARS-CoV-2 VLPs can be efficiently produced in insect cells and purified using scalable affinity chromatography.

## Introduction

Virus-like particles (VLPs) are multiprotein capsids structurally and functionally resembling infectious virions. They are formed by structural viral proteins with inherent property for self-assembly when overexpressed in a suitable host cell. These capsids not only mimic the morphology of the parental virus but also can specifically transduce permissive cells. Importantly, VLPs lack the viral genome, which makes them safe and valuable tools in several research areas. In particular, VLPs can be used for vaccine development and studies on virus–host interactions. The use of such virus substitutes is especially beneficial when high-risk viruses are under investigation, as working with VLPs does not require special safety measures.

The severe acute respiratory syndrome coronavirus 2 (SARS-CoV-2) is the causative agent of coronavirus disease 2019 (COVID-19), which is currently the major health, economic, and social problem worldwide. Like other coronaviruses, the SARS-CoV-2 comprises the core consisting of the viral RNA tightly associated with the nucleocapsid (N) protein, protected by the lipid envelope decorated with the structural proteins. These include the spike (S) glycoprotein, the key element interacting with the receptor and responsible for the virus entry into target cells; the membrane (M) protein forming the curvature of the virion and linking all the elements; and the envelope (E) protein important for the viral assembly and budding.

Previous studies have shown that the co-expression of structural coronaviral proteins in eukaryotic cells results in VLP assembly and release (Vennema et al., 1996; Mortola and Roy, 2004; Liu et al., 2013; Wang et al., 2017a,b). Successful production of coronavirus-like particles has been reported for SARS-CoV (Ho et al., 2004; Mortola and Roy, 2004; Hsieh et al., 2005; Nakauchi et al., 2008; Siu et al., 2008), mouse hepatitis virus (MHV) (Vennema et al., 1996; de Haan et al., 1998; Narayanan et al., 2000; Boscarino et al., 2008; Arndt et al., 2010), avian infectious bronchitis virus (IBV) (Corse and Machamer, 2000), porcine transmissible gastroenteritis virus (TGEV) (Baudoux et al., 1998; Gelhaus et al., 2014), and porcine epidemic diarrhea virus (PEDV) (Wang et al., 2017a).

Recently, production of SARS-CoV-2 originating VLPs has been reported as well. Described particles can be divided into several subtypes, depending on the technology used: homotypic VLPs composed of coronaviral proteins only (Boson et al., 2020; Lu et al., 2020; Plescia et al., 2020; Swann et al., 2020; Xu et al., 2020; Kumar et al., 2021; Sharma et al., 2021); heterotypic (chimeric) VLPs, where the SARS-CoV-2 S protein is incorporated into a heterologous viral scaffold (animal viruses or bacteriophages) (Fougeroux et al., 2021; Roy et al., 2021; Tan et al., 2021); and heterotypic VLP where no coronaviral proteins are present, but fragments of coronaviral genetic material are encapsulated in either plant viruses or bacteriophages and may be used, for example, for diagnostic purposes (Chan et al., 2021). Some authors also classify pseudoviruses displaying SARS-CoV-2 S protein as VLPs (Ju et al., 2021).

Almost all homotypic SARS-CoV-2 VLPs described so far were produced in the mammalian hosts (mainly HEK 293T and Vero cells). However, these producer lines yield relatively low amounts of VLPs, which may be a limitation for further use and upscaling. Similarly, the most frequently used purification method – sucrose density gradient ultracentrifugation – is not suitable for large-scale purification and thus remains as another limiting factor in the high-throughput production of VLPs.

Here, we show that functional SARS-CoV-2 VLPs may be efficiently produced in insect cells using the baculovirus (BV) expression system. Taking advantage of the structural proprieties of the M protein, we show that these particles can be easily purified and concentrated using scalable and straightforward affinity chromatography. Additionally, we developed tools for quantifying and characterizing the obtained particles by nanoparticle tracking analysis (NTA), dynamic light scattering (DLS), and transmission electron microscopy (TEM). Finally, we show that obtained particles are functional, as they transduce cells permissive for SARS-CoV-2 infection, using the same receptor as the infectious virus.

## Materials and Methods

### Cell Lines and Viruses

Sf9 (*Spodoptera frugiperda*, ATCC: CRL-1711) and HF (High Five, *Trichoplusia ni*, ATCC: CRL-7701) cells were cultured in ESF (Expression Systems, Davis, CA, United States) medium supplemented with 2% fetal bovine serum (FBS) (Thermo Fisher Scientific, Warsaw, Poland), 100 μg/ml of streptomycin, 100 IU/ml of penicillin, 10 μg/ml of gentamycin, and 0.25 μg/ml of amphotericin B. The culture was maintained in humidified incubator at 27°C. Sf9 cells were used for BV generation and amplification, while HF cells were used for recombinant proteins expression.

A549 (ATCC: CCL-185; human lung carcinoma cell line) and Vero (*Cercopithecus aethiops*; kidney epithelial; ATCC: CCL-81) were maintained in Dulbecco’s Modified Eagle Medium (DMEM; Corning 10-017-CV, Warsaw, Poland) supplemented with 5% FBS 100 μg/ml of streptomycin and 100 IU/ml of penicillin. The culture was maintained at 37°C under 5% CO_2_. A549 cells with angiotensin-converting enzyme 2 (ACE2) overexpression (A549^ACE2+^) (Milewska et al., 2014) were cultured in the same manner with supplementation with G418 (5 mg/ml; BioShop, Burlington, ON, Canada).

### Genetic Constructs (Plasmids and Bacmids)

The codon-optimized (for insect expression) genes encoding for 6xHis-M, E, N, and S-HA (YPYDVPDYA) proteins were synthesized (GeneArt, Thermo Fisher Scientific, Erlangen, Germany), delivered in pMA plasmids and subcloned to pFastBac Dual plasmids (Thermo Fisher Scientific, Poland). As monocistronic and bicistronic pFastBac plasmids were created, the following denotation was adopted in the later text: (M + E) and (His-M + E) for bicistronic vectors. Recombinant bacmids and BVs were generated using BAC-TO-BAC system (Thermo Fisher Scientific, Poland). Briefly, *Escherichia coli* DH10-Bac competent cells were transformed with recombinant pFastBac Dual vectors, and the isolated bacmid DNA was purified and transfected into Sf9 cells. After 6 days, recombinant BVs (rBVs) were harvested, amplified, and titrated using plaque assay method.

### Theoretical Protein Topology Prediction

Membrane (M) protein topology prediction was generated with www.cbs.dtu.dk/services/TMHMM online tool.

### Sodium Dodecyl Sulfate–Polyacrylamide Gel Electrophoresis and Western Blotting

Insect cells or culture media were harvested and resuspended in denaturing buffer containing 8% sodium dodecyl sulfate (SDS) and 400 mM of β-mercaptoethanol and boiled for 5 min (unless indicated otherwise). Samples were resolved by 10–12% Laemmli SDS–polyacrylamide gel electrophoresis (SDS-PAGE). PageRuler Prestained Protein Ladder (Thermo Fisher Scientific, Poland) was used in this study as protein size marker. Gels were subjected to electrotransfer in 25 mM of Tris, 192 mM of glycine, and 20% methanol buffer onto the activated polyvinylidene difluoride (PVDF) membrane. The membrane was blocked with 5% skim milk in Tris-buffered saline supplemented with 0.05% of Tween 20, followed by 1-h incubation with mouse anti-His tag antibody (1:2,000; Thermo Fisher Scientific, Poland); rabbit polyclonal anti-N SARS-CoV-2 serum (1:10,000; a kind gift of Dr. Beata Gromadzka) or rabbit polyclonal anti-S SARS-CoV-2 serum (1:1,000, Protein Sci) and, respectively, anti-mouse (1:20,000, Dako, Glostrup, Denmark) and anti-rabbit (1:20,000, Dako, Denmark) secondary antibodies conjugated with horseradish peroxidase (HRP). The signal was developed using a Pierce ECL Blotting Substrate (Thermo Fisher Scientific) and visualized in a Bio-Rad ChemiDoc detector (Bio-Rad Laboratories, Hercules, CA, United States).

### Confocal Microscopy

For transduction of VLPs into target cells, Vero E6 cells or wild-type A549 cells or A549 expressing ACE2 receptor was grown to 80% confluence for 48 h in 12-well culture plates on glass coverslips. Cells were then washed with phosphate-buffered saline (PBS) and inoculated with 300 μl of VLP-containing supernatants collected 72 h post infection from HF cultures. Next, Vero E6, A549 ± E ACE2 cells were incubated for 2.5 h at 37°C under 5% CO_2_ and further washed thrice with PBS. Subsequently, cells were fixed with 4% formaldehyde, permeabilized with 0.2% Triton X-100 in PBS, and blocked for 1 h with 5% bovine serum albumin in PBS. As anti-His antibody was not suitable for in cell protein staining, and thus His-M protein could not be detected, we used anti-M and anti-HA antibodies to detect His-M and S-HA-His protein in transduced cells. Rabbit polyclonal anti-M antibody (1:1,000, ProSci, United States) purchased from antibodies-online.com, catalog nb: ABIN6952906 and mouse monoclonal anti-HA antibody (1:500, Antibodies Online, Limerick, PA, United States) and secondary anti-rabbit and anti-mouse antibodies conjugated with Alexa 488 (1:400, Invitrogen, Carlsbad, CA, United States) were used. Cell nuclei were stained with DAPI (0.1 μg/ml in PBS; Sigma-Aldrich, Poznań, Poland). Additionally, actin filaments were visualized with phalloidin conjugated with Alexa 647 (0.132 μM, Sigma-Aldrich, Poland). Coverslips were mounted on glass slides with Prolong Diamond (Sigma-Aldrich, Poland).

Fluorescent images were acquired under a Zeiss LSM 880 confocal microscope (Carl Zeiss Microscopy GmbH, Oberkochen, Germany) with 40 × 1.4 NA oil immersion objective. Images were acquired using ZEN 2012 SP1 software (Carl Zeiss Microscopy GmbH, Germany). All images were processed using ImageJ 1.53c (Schindelin et al., 2012) (National Institutes of Health, Bethesda, MD, United States).

### Protein Purification

HF cells were infected with (His-M + E) BV at multiplicity of infection (MOI) = 4 and optionally S BV at MOI = 1 and cultured for 72 h. VLPs in secreted fraction were harvested by centrifugation (1,000 × *g*, 10 min) of cell culture suspension. The supernatant was diluted 1:1 (v/v) with 50 mM of Tris, pH 7.9, 150 mM of NaCl, and 20 mM of imidazole (binding buffer) and incubated for 30 min at room temperature with agarose beads coupled with Ni^2+^-bound nitrilotriacetic acid (His-Pur Ni-NTA, Thermo Fisher Scientific, Poland) preequilibrated in the binding buffer. After three washes of the resin (with binding buffer), the protein was eluted with 50 mM of Tris, pH 7.9, 150 mM of NaCl, and 300 mM of imidazole (elution buffer). Collected fractions were analyzed for the presence for the His-tag (M protein) and S protein using Western blotting, as described above. Fractions containing both proteins (presumably His-tagged VLPs) were pooled and dialyzed against PBS to remove imidazole, using Pur-A-Dialyzer (Sigma Aldrich, Poland). Purified VLPs were used for DLS and NTA measurements and TEM analysis.

### Dynamic Light Scattering

Purified VLPs were analyzed using DLS. Hydrodynamic particle size measurements were done in ZEN2112 microcuvettes at 25°C using Zetasizer Nano S DLS instrument (Malvern Instruments, Malvern, United Kingdom). Light scattering was measured 15 times at 10-s intervals for each sample. The data were analyzed using Zetasizer ver.7.11 software (Malvern Instruments, United Kingdom).

### Nanoparticle Tracking Analysis

Concentration and size distribution of isolated SARS-CoV-2 VLPs (His-M + E and His-M + E + S) were analyzed using NTA-based NanoSight NS300 analyzer (Malvern Panalytical, United Kingdom). Before the measurement, VLP samples were diluted in 0.2-μm-filtered DPBS (Lonza) to reach optimal particle concentration suitable for instrument measurement range. For VLP quantification in time, His-M + E and His-M + E + S samples were divided into five portions each, directly after purification. One portion was immediately used for NTA measurement (day 0), two portions were stored in the fridge (4°C), and two portions were stored in the freezer (-20°C), without any additives. Stored portions were analyzed after 7 and 14 days, without freeze–thawing cycles. Each sample was measured with three 60-s tracking repetitions in syringe pump flow mode, using camera level of 12. Subsequently, tracking data were analyzed with threshold parameter set on 2. Average particle concentration as well as size distribution was calculated using NTA Software ver. 3.4 (Malvern Panalytical).

### Electron Microscopy

Purified VLPs were fixed in 2.5% paraformaldehyde in cacodylate buffer and inoculated onto single-hole copper grids coated with a support film (Formvar 15/95E, Sigma-Aldrich, St. Louis, MO, United States). After being dried, the material was stained with uranyl acetate (Polyscience, Inc., Warrington, PA, United States) and citrate lead (Sigma-Aldrich, St. Louis, MO, United States). Subsequently, grids were washed with water and dried in air at room temperature. The ultrastructural observations were performed by TEM FEI Tecnai G2 BioSpirit, at an accelerating voltage of 120 kV. Electron microscopic examinations were performed in the Electron Microscopy Laboratory, Department of Histology and Cell Pathology in Zabrze, Medical University of Silesia in Katowice.

## Results

### Production of His-Tagged Severe Acute Respiratory Syndrome Coronavirus 2 Virus-Like Particles

**FIGURE 1:**
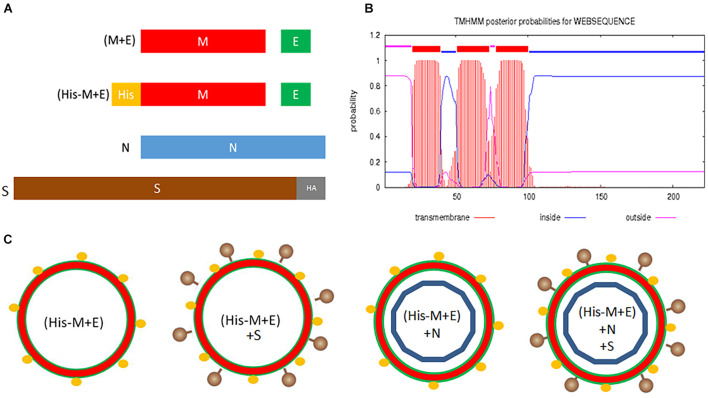
Design of SARS-CoV-2 VLPs. **(A)** Genetic constructs (pFastBac Dual plasmids) coding for structural proteins of SARS-CoV-2: (M + E) and (His-M + E) are bicistronic vectors; N and S-HA are monocistronic vectors. **(B)** Predicted topology of the M protein. **(C)** Possible VLPs resulting from co-infection of insect cells with prepared vectors. Legend: M, membrane protein; E, envelope protein; N, nucleocapsid protein; S, spike protein; SARS-CoV-2, severe acute respiratory syndrome coronavirus 2; VLPs, virus-like particles.

SARS-CoV-2 VLPs were produced using a BV expression system by adopting our previously optimized protocol for HCoV-NL63 VLPs (Naskalska et al., 2018). rBVs coding for M and E proteins (bicistronic), N protein, and HA-tagged S protein (both monocistronic) were created. Additionally, we engineered a bicistronic BV coding for the His-tagged M protein and E protein (Figure 1A). The His-tag location was designed based on the theoretical prediction of the M protein topology, indicating that the N-terminal part of this protein is exposed at the surface of the virion (Figures 1B,C).

**FIGURE 2:**
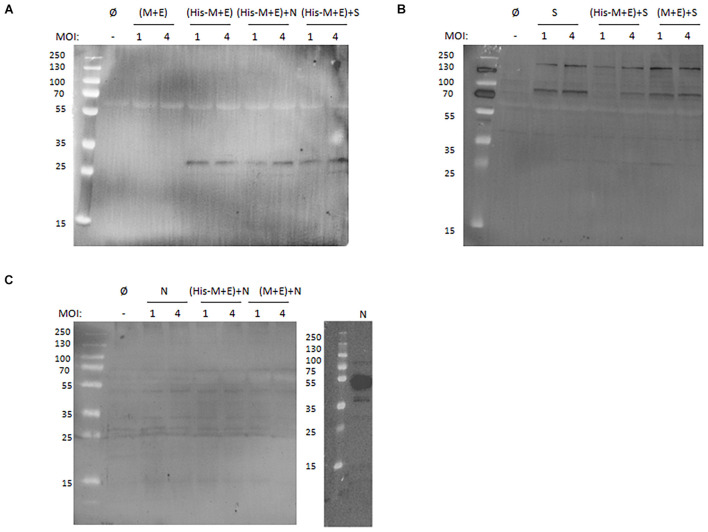
Western blotting analysis of SARS-CoV-2 proteins secreted from insect cells. **(A)** Anti-His antibody detection of culture media collected from cells infected with (M + E) BV and (His-M + E) BV alone or in presence with N BV or S BV. **(B)** Anti-S antibody detection of culture media collected from cells infected with S BV alone or in the presence of (His-M + E) BV and (M + E) BV. **(C)** Anti-N antibody detection of culture media collected from cells infected with N BV alone or in the presence of (His-M + E) BV and (M + E) BV (left) and cell lysate from cells infected with N BV (right). In each case, MOI = 1 and MOI = 4 were tested. “Ø” denotes negative control (medium collected from uninfected insect cells). SARS-CoV-2, severe acute respiratory syndrome coronavirus 2; BV, baculovirus; MOI, multiplicity of infection.

For the enveloped coronavirus-like particles, it is generally accepted that the presence of M, E, and N proteins in the culture medium harvested from the producer cells indicates efficient assembly and release of the putative VLPs (Vennema et al., 1996; Mortola and Roy, 2004; Liu et al., 2013; Wang et al., 2017b). To verify this in our experimental setting, culture media from HF cells infected with the generated BVs in different combinations and using a different MOI were tested. As shown in Figure 2A, the His-M protein can be detected with anti-His antibody when expressed solely from His-M + E BV (MOI = 4) and after co-infection with N or S-HA BVs. This indicates that the protein release from producer cells (and likely VLP formation) is not affected by the His-tag and does not depend on N and S proteins. This finding is consistent with our previous observation that co-expression of M and E is sufficient for secretion (and assembly) of VLPs (Naskalska et al., 2018). Interestingly, the S-HA protein can be detected in the culture medium when expressed alone or co-expressed with (His-M + E) or (M + E) (Figure 2B). By contrast, N protein could not be detected when expressed either alone or with (His-M + E) or (M + E), indicating that N protein is not released from insect cells, even though produced (Figure 2C).

### The Functionality of His-Tagged Severe Acute Respiratory Syndrome Coronavirus 2 Virus-Like Particles

**FIGURE 3:**
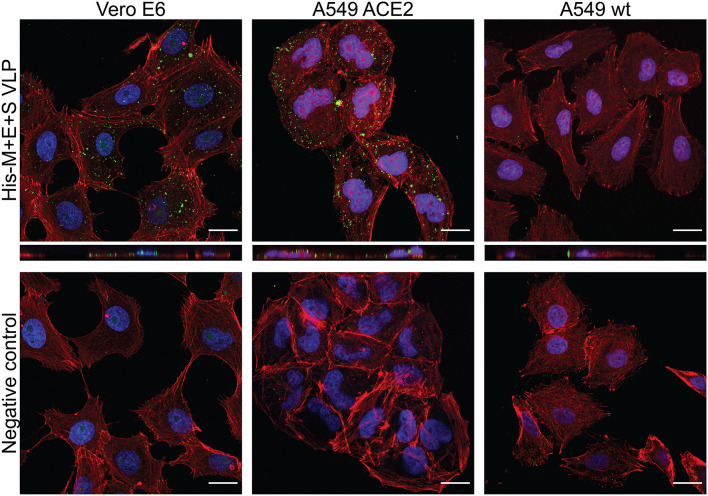
SARS-CoV-2 VLP entry to target cells. His-M + E + S VLPs were incubated with Vero cells (left panel), A549 expressing ACE2 receptor cells (middle panel), and wild-type A549 cells (right panel). VLP entry was visualized by M protein (in Vero cells) or S protein (in A549 cells) detection with respective antibodies (shown in green); nuclei are stained with DAPI (blue), and actin filaments are stained with fluorescently labeled phalloidin (shown in red). Controls are images of respective cells incubated with mock samples (collected from uninfected insect cells) and stained identically as cells incubated with VLPs. SARS-CoV-2, severe acute respiratory syndrome coronavirus 2; VLP, virus-like particle; ACE2, angiotensin-converting enzyme 2.

To investigate whether obtained VLPs possess the capacity to enter cells carrying an appropriate receptor, their internalization to Vero cells was investigated. Vero E6 cells naturally express the ACE2 and are susceptible to the SARS-CoV-2 infection (Wrapp et al., 2020). Additionally, A549 cell line engineered to express the ACE2 receptor and wild-type A549 cell line was compared, in order to eliminate the possibility of unspecific VLP uptake. Target cells were seeded on slides and incubated with media harvested from insect cells expressing His-M + E + S VLPs and mock media (harvested from uninfected insect cells). Target cells were then fixed and stained for analysis in a fluorescence confocal microscope. VLPs were detected with anti-M and anti-HA (for S detection) antibodies, as described in the “Materials and Methods” section. As shown in Figure 3, VLPs could be observed only in ACE2-expressing cells, indicating the receptor-dependent entry.

### Purification and Characterization of His-Tagged Severe Acute Respiratory Syndrome Coronavirus 2 Virus-Like Particles

**FIGURE 4:**
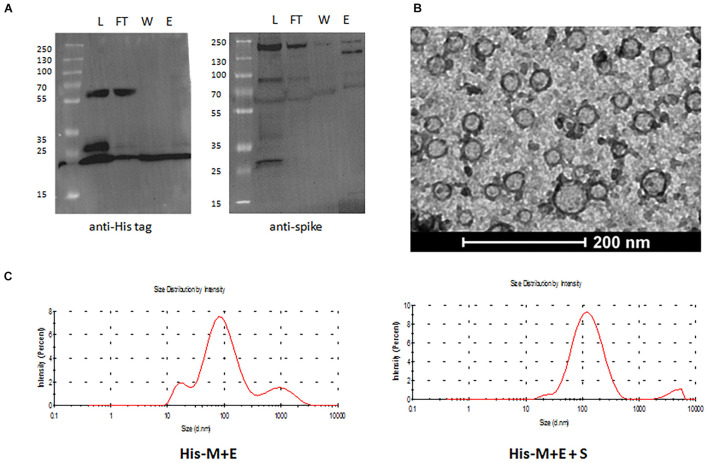
Purification and characterization of His-tagged SARS-CoV-2 VLPs. **(A)** Western blotting analysis of fractions eluted from Ni-NTA purification: L, load; FT, flow through; W, wash; E, elution. Proteins on the left membrane were detected with anti-His antibody (reflecting presence of the His-M protein), whereas on the right are membrane with anti-S antibody. **(B)** TEM image of His-M + E VLPs. **(C)** DLS measurements of mean hydrodynamic diameter of His-M + E (left) and His-M + E + S (right) VLPs. SARS-CoV-2, severe acute respiratory syndrome coronavirus 2; VLP, virus-like particle; NTA, nitrilotriacetic acid; TEM, transmission electron microscopy; DLS, dynamic light scattering.

In order to assess if His-tagged VLPs can be purified using affinity chromatography, samples containing His-M + E VLPs with and without S were incubated with Ni-NTA agarose and further eluted with a buffer containing imidazole. Collected fractions were analyzed by Western blotting with anti-His and anti-S antibodies (Figure 4A). This result provides evidence that His-tagged VLPs were bound to the Ni-coupled resin, supporting the assumption that N-terminus of the M protein is displayed on the viral particle and can be used for appending His-tag (or other peptides).

To verify the proper assembly and integrity of the produced VLPs, samples containing purified particles were examined using TEM. Images show spherical particles of diameter ranging from 30 to 100 nm (Figure 4B and Supplementary Figure 1), which is consistent with the reported size of SARS-CoV-2 (ca. 100 nm) diameter. Next, DLS was used to measure the mean hydrodynamic diameter of particles in solution, which was shown to be 115 nm for His-M + E VLPs and 137 nm for His-M + E + S VLPs (Figure 4C). Noteworthy, both TEM and DLS analyses revealed that obtained particles are not homogeneous in terms of their size. This issue has been further addressed using NTA (described in the next section).

### Quantification and Stability of His-Tagged Severe Acute Respiratory Syndrome Coronavirus 2 Virus-Like Particles

**FIGURE 5:**
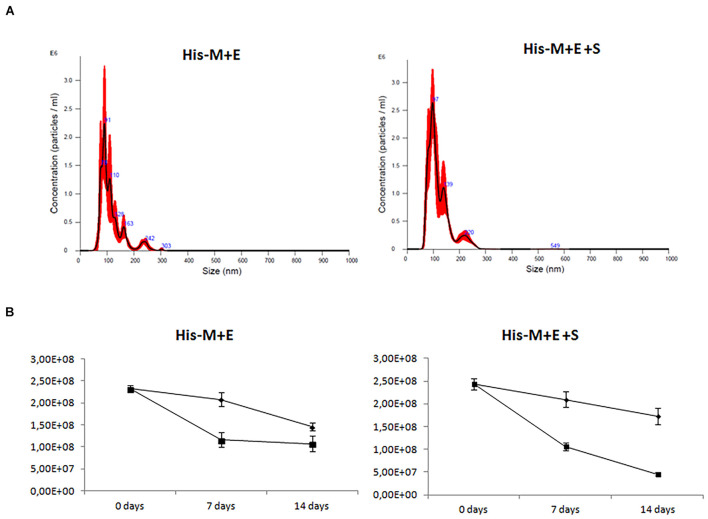
Size distribution and quantification of His-tagged SARS-CoV-2 VLPs. Nanoparticle tracking analysis (Steppert et al., 2017). **(A)** Representative histograms showing the average size distribution of measured particles. Numbers in blue indicate particular peaks’ size, whereas the red surface corresponds to the standard deviation value. **(B)** Concentration (per 1 ml) of particles directly after purification, after 7 and 14 days’ storage in the fridge (4°C: diamonds) and freezer (−20°C: squares). Numbers shown on the y-axis are crude results from samples diluted 10 × before measurement. SARS-CoV-2, severe acute respiratory syndrome coronavirus 2; VLP, virus-like particle.

Nanoparticle tracking analysis was used to assess the concentration and size distribution of particles in the purified sample. The estimated number of VLPs purified from 10 ml of culture is 3.8 × 10^9^ for His-M + E and 5.8 × 10^9^ for His-M + E + S, which means that potentially 3.8 × 10^11^ to 5.8 × 10^11^ particles can be obtained from 1 L of culture of insect cells. Based on NTA measurement, mean particle size in His-M + E and His-M + E + S VLPs samples was calculated as 114.0 ± 2.7 and 120.3 ± 1.5 nm, respectively (Figure 5A), which is in accordance with DLS results.

Finally, NTA was used to investigate VLP stability when stored in 4°C and −20°C for 7 and 14 days, respectively. The concentration of His-M + E and His-M + E + S VLPs was assessed immediately after purification and after storage at 4°C or at −20°C. When samples were stored at 4°C, a 10–14% decrease in particle number was observed after 7 days and 30–37% after 14 days (Figure 5B and Supplementary Table 1). A 50–57% decrease was recorded after 7 days and 54–82% after 14 days when samples were stored at −20°C; however, samples were frozen without cryoprotectants.

## Discussion

In this manuscript, we show that SARS-CoV-2 VLPs can be efficiently produced in insect cells, which are generally considered as more robust and cost-effective producer cells than mammalian hosts. Previous studies report expression of SARS-CoV-2 VLPs in 293T and Vero cell lines (Boson et al., 2020; Plescia et al., 2020; Swann et al., 2020; Xu et al., 2020; Sharma et al., 2021), and one recent study in insect cells (Mi et al., 2021); however, the authors do not specify the yield. It has to be noted that the glycosylation pattern of proteins expressed in insect cells differs from that of the native form produced in human cells, which is important for viral surface glycoproteins, as they play important roles in virus–host recognition and immune recognition and evasion. The major target for neutralizing antibodies – the spike protein – is indeed highly glycosylated when expressed in mammalian cells (Ke et al., 2020; Watanabe et al., 2020). However, it has been demonstrated that insect – expressed S protein (as well as insect – expressed S1, S2, RBD fragments) – not only reacted with the convalescent sera from patients infected with SARS-CoV-2 but also elicited high neutralization IgG titers in immunized animals, suggesting that these proteins maintain the native-like SARS-CoV-2 epitopes (Li et al., 2020). It is also worth noticing the key regions of the spike protein: the receptor binding domain and heptapeptide repeat sequences (HR1 I HR2) are not glycosylated (Ma et al., 2020).

In this work, BV-based expression vectors were designed to bear genes of both structural proteins forming the capsid M and E proteins, as we previously observed that such a design improves the particle assembly efficiency (Naskalska et al., 2018). N and S proteins were delivered on separate vectors to enable manipulation of composition to ensure best assembly parameters. However, it turned out that N protein is not incorporated into particles when co-expressed with M and E from different vectors. By contrast, tricistronic construct (M and E and N) resulted in assembly and release of VLPs composed of all three proteins (Supplementary Figure 2 and Supplementary Table 2). Noteworthy, the minimal protein requirement to form these particles is disputable. Finding that N protein is dispensable for SARS-CoV-2 VLP formation is in accordance with Swann et al. (2020) and Lu et al. (2020) but in contrast to Boson et al. (2020) and Plescia et al. (2020). It could be that N protein co-expression plays an additive role in the VLP egress, as reported by Kumar et al. (2021). In our experimental setting, S protein was detectable in the insect cell culture medium, both when expressed alone and when co-expressed with M (or His-M) and E proteins. To ensure that S protein is actually incorporated into particle in the latter case, we demonstrated that S protein was detectable in fractions eluted from Ni^2+^-coupled resin and thus anchored to His-tagged M protein (Figure 4A).

Importantly, we have shown that our VLPs can specifically enter ACE2-expressing cells. This provides evidence that obtained VLPs are functional in terms of transducing target cells. By using appropriate control, we show that the observed cell entry is receptor-dependent and thus specific (Figure 3 and Supplementary Figure 2). Moreover, efficient and observable transduction was achieved as a result of simple incubation (2.5 h, 37°C, 5% CO_2_) of target cells with VLPs, which is identical to the protocol used for *in vitro* SARS-CoV-2 cell infection. So far, SARS-CoV-2 VLP internalization to target cells has been reported by Kumar and co-workers (Kumar et al., 2021), who used a newly developed NanoBiT technology involving a luciferase-based assay to quantify VLPs internalized to the cell. We believe that our traditional approach (laser scanning confocal microscopy imaging) provides complementary insight into understanding cell entry process. Plescia and co-workers also declare that they observe SARS-CoV-2 entry to target cells (Plescia et al., 2020), but these authors use spinoculation to enforce endocytosis.

It was demonstrated that our VLPs efficiently and specifically entering cells expressing ACE2 is of importance, because as they mimic the native virus in this aspect, they will likely activate similar components of the immune response. For instance, when administrated intranasally – the natural infection route for SARS-CoV-2 infection – one may expect that these VLPs will elicit IgA antibodies. Notably, it was found that IgA-mediated mucosal immunity could be the most critical defense mechanism against SARS-CoV-2 and may reduce viral shedding and transmission of the virus from person to person (Butler et al., 2020; Sterlin et al., 2021). Also, VLPs entering the cells are processed and further presented in the context of the major histocompatibility complex, thus potentially triggering better cellular immune response (Lukacs and Malinczak, 2020). This has important implications when considering the development of a vaccine for protection against COVID-19.

All previous reports describe SARS-CoV-2 VLP purification using density gradient ultracentrifugation, which is a tedious and poorly scalable procedure. Here, we provide evidence that SARS-CoV-2 VLPs can be simply purified using affinity chromatography technique. To this end, we took advantage of the theoretical topology of the M protein, predicting that its N-terminal part protrudes from the particle. Indeed, fusing His-tag allowed for binding the Ni^2+^-coupled resin and further elution of purified particles. Appending peptide tags to SARS-CoV-2 proteins has been already demonstrated by Xu et al. (2020), but these authors inserted the FLAG tag to the C-terminus of the M (and S, N, and E) proteins, which most likely result in the tag being locked inside the particle. Additionally, we assessed the amount of contaminating baculoviral particles in the purified preparations of our insect that expressed VLPs (Supplementary Figure 4 and Supplementary Table 3). We believe that these residual BVs could be removed by additional purification column, such as Capto Q (Cytiva, Marlborough, MA, United States).

Our particles were morphologically characterized by TEM and DLS and quantified by NTA. This may be of special interest for researchers developing diagnostic tools and using VLPs as substitutes to infectious virions, as the number of particles is essential for setting the detection threshold. We have also addressed the stability of purified VLPs. Similarly to other coronavirus-like particles, SARS-CoV-2 VLPs are relatively unstable, at least in tested conditions (Sharma et al., 2021). This could be likely improved by adding a cryoprotectant or stabilizing agents and is currently under our investigation. Additionally, we expect that incorporation of the N protein into SARS-CoV-2 VLPs would improve their stability (de Haan et al., 1998; Arndt et al., 2010; Kumar et al., 2021).

## Conclusion

In this work, we show that functional SARS-CoV-2 VLPs can be successfully produced using insect cells. Importantly, obtained particles retain the ability of their parental virus to specifically enter cells expressing the ACE2 receptor. Additionally, by demonstrating that His-tag fusion to the N-terminus of the M protein results in properly assembled particles that can be easily purified using affinity chromatography, we provide a method for the scale-up of SARS-CoV-2 VLP production. It is worth noting that SARS-CoV-2 VLPs are an attractive alternative to the commonly used pseudoviruses (such as lentivirus-based systems), especially to study virus–host interactions or virus-induced immune responses, as in VLPs, viral structural proteins are present in their natural context. In conclusion, we believe that obtained SARS-CoV-2 VLPs could serve in multiple applications, such as diagnostic tools and vaccine production or drug development, thereby contributing to global effort in conquering the pandemic.

## Data Availability Statement

All relevant data is contained within the article. Further inquiries can be directed to the corresponding authors.

## Author Contributions

AN, AS, AD, KJ, and BG: investigation. AN and KP: conceptualization, funding acquisition, writing – original draft, and supervision. All authors writing, review, and editing.

## Conflict of Interest

BG was employed by company NanoExpo®. The remaining authors declare that the research was conducted in the absence of any commercial or financial relationships that could be construed as a potential conflict of interest.

## Publisher’s Note

All claims expressed in this article are solely those of the authors and do not necessarily represent those of their affiliated organizations, or those of the publisher, the editors and the reviewers. Any product that may be evaluated in this article, or claim that may be made by its manufacturer, is not guaranteed or endorsed by the publisher.
