# Supplementary material for: Functional Severe Acute Respiratory Syndrome Coronavirus 2 Virus-Like Particles From Insect Cells
Source: Front Microbiol. 2021 Oct 20;12:732998. doi: 10.3389/fmicb.2021.732998 (PMC8565087; doi:10.3389/fmicb.2021.732998)
Supplement: Supplementary file 1 [file Data_Sheet_1.docx]

Supplementary Material


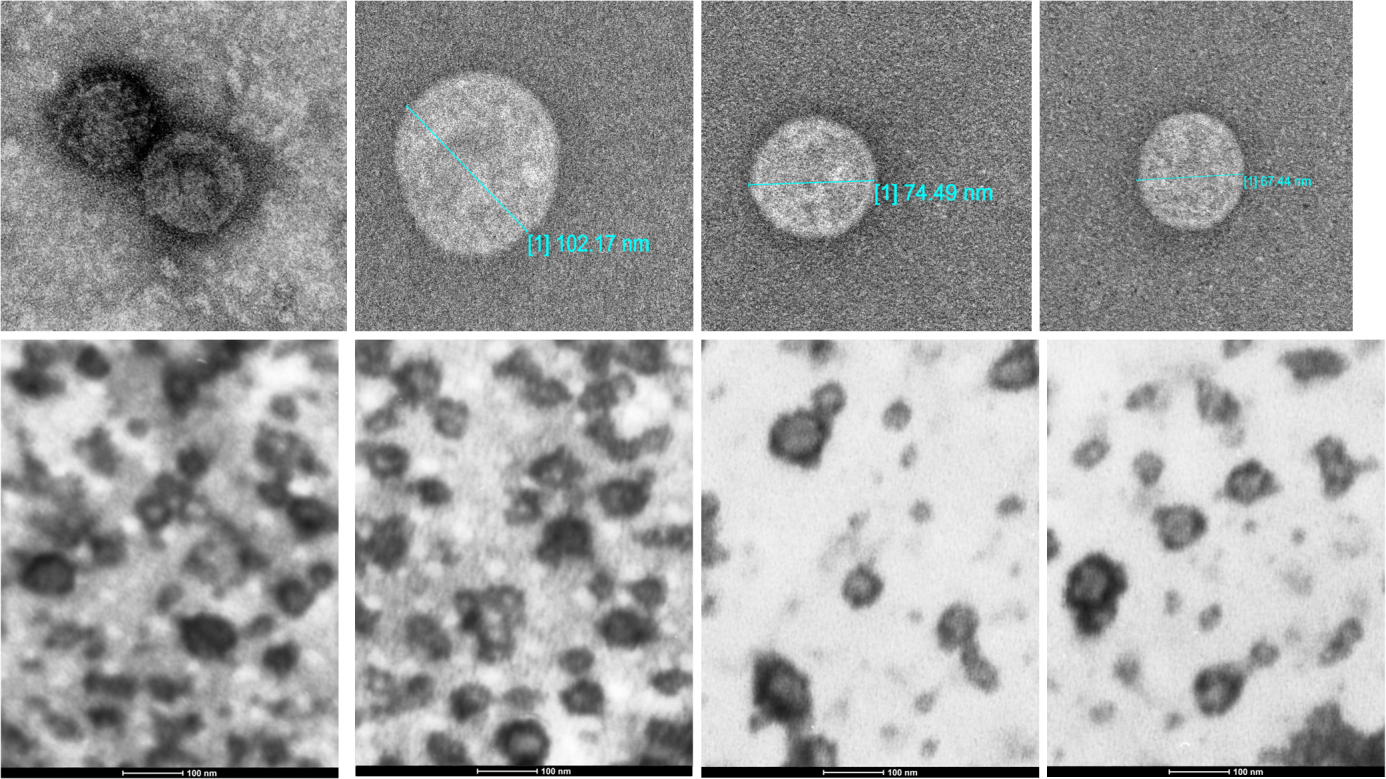


**Supplementary Figure 1.TEM images of SARS-CoV-2 VLP.**TEM images of negatively stained His-M+E VLPs (upper panel) and positively stained His-M+E+S VLPs (lower panel).

*
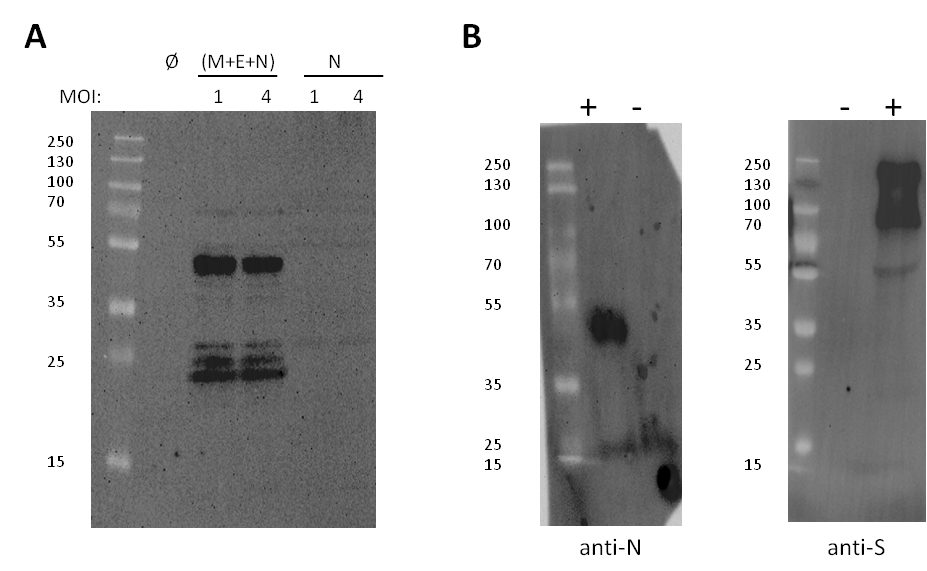
*

**Supplementary Figure 2. Western blot analysis of SARS-CoV-2 N protein in M+E+N VLPs. A** Western blot analysis (with anti-N antibody detection) of culture media collected from cells infected with (M+E+N) tricistronic BV and N BV. MOI 1 and 4 were tested. “Ø” denominates negative control (medium collected from uninfected insect cells). **B** Control Western blot analysis (with anti-N and anti-S antibodies detection) of samples containing heat-inactivated SARS-CoV-2 (harvested from infected Vero cells, “+”) demonstrating specificity of antibodies used in this study. “-” corresponds to medium sample collected from uninfected Vero cells.

*
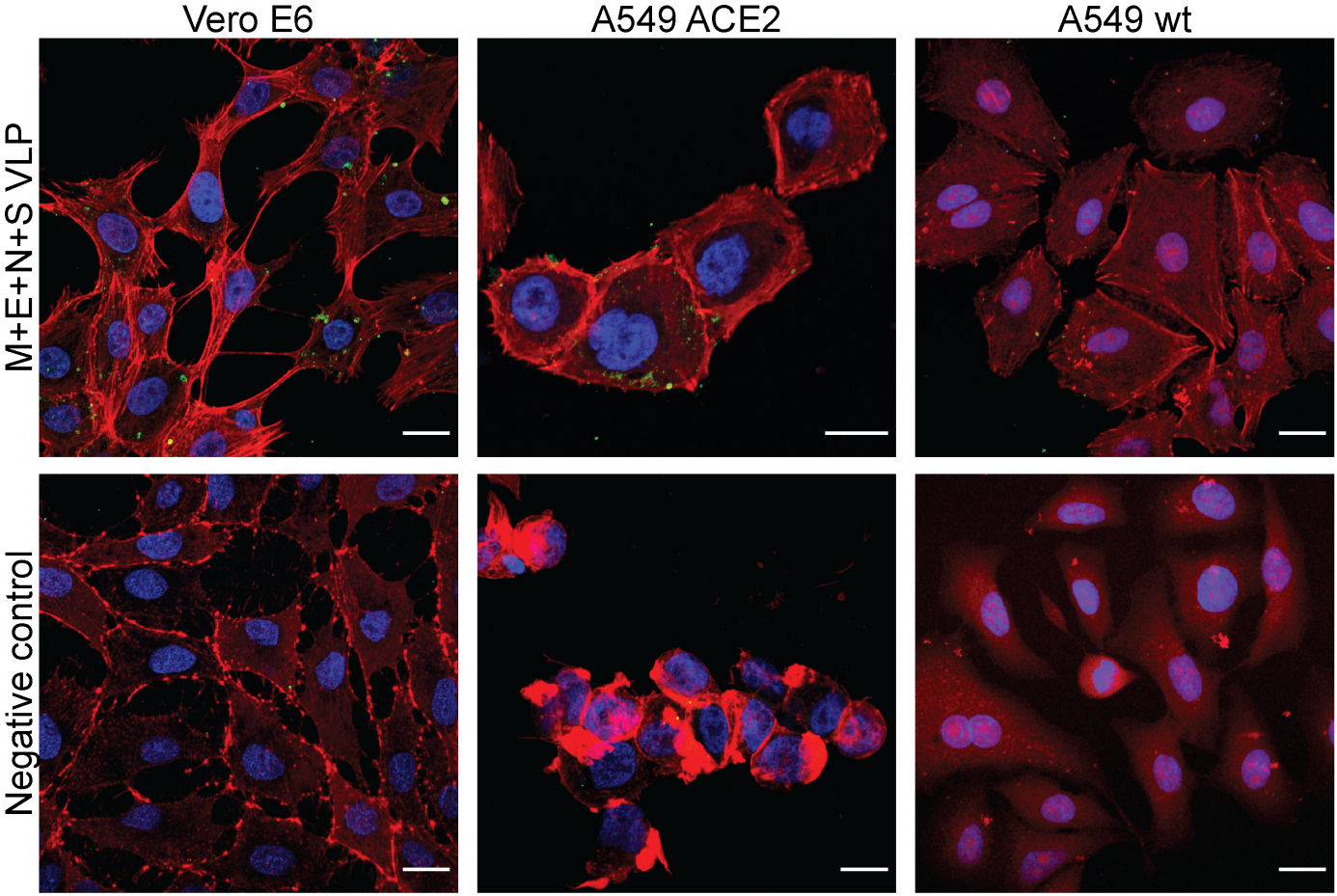
*

**Supplementary Figure 3. SARS-CoV-2 VLP entry to target cells.** M+E+N+S VLPs were incubated with Vero cells (left panel), A549 cells expressing ACE2 receptor (middle panel) and wild-type A549 cells (right panel). VLP entry was visualized by N protein detection with anti-N polyclonal rabbit serum (shown in green); nuclei are stained with DAPI (blue), and actin filaments are stained with fluorescently labeled phalloidin (shown in red). Controls are images of respective cells incubated with mock samples (collected from uninfected insect cells) and stained identically as cells incubated with VLPs. Scale bar 20 µm.

**Table S1.** Number of SARS CoV-2 VLPs suspended in 1 mL of PBS buffer, purified from 25 mL culture media collected from insect cells and diluted 10x before measurement.

|  | **fresh** | **7 days** | change | **14 days** | change |
| --- | --- | --- | --- | --- | --- |
| His-M+E 4°C | 2.32e+08 +/- 8.33e+06 | 2.08e+08 +/- 1.50e+07 | 10.4% ↓ | 1.46e+08 +/- 8.70e+06 | 37.1% ↓ |
| His-M+E -20°C |  | 1.16e+08 +/- 1.65e+07 | 50% ↓ | 1.07e+08 +/- 1.73e+07 | 53.88% ↓ |
| His-M+E+S 4°C | 2.44e+08 +/- 1.22e+07 | 2.10e+08 +/- 1.63e+07 | 14% ↓ | 1.73e+08 +/- 1.74e+07 | 30% ↓ |
| His-M+E+S -20°C |  | 1.06e+08 +/- 8.91e+06 | 56.6% ↓ | 4.58e+07 +/- 4.44e+06 | 81.3% ↓ |

**Table S2.** Summary of Western blot analysis of protein released to culture medium by insect cells infected with respective BVs.

| Recombinant BVs used to infect insect cells for expression | **M** protein detected in culture medium | **N** protein detected in culture medium | **S** protein detected in culture medium |
| --- | --- | --- | --- |
| (His-M+E) | + (anti-His) |  |  |
| (His-M+E) and N | + (anti-His) | - |  |
| (M+E) and N |  | - |  |
| N |  | - |  |
| (His-M+E) and S | + (anti-His) |  | + |
| (M+E) and S |  |  | + |
| S |  |  | + |
| (M+E+N) |  | + |  |
| (M+E+N) and S |  | + | + |

In order to assess the amount of residual baculovirus particles in purified VLP samples, a Western blot analysis for detection of gp64 protein – a structural protein of baculoviruses – was performed.

First, we compared the crude media harvested from insect cells expressing VLPs with the purified VLPs. For this we expressed and purified His-M+E and His-M+E+S VLPs as described in the manuscript. Additionally, we expressed and identically treated beta-glucuronidase (gus)/chloramphenicol acetyl transferase (cat) proteins (as positive baculovirus control) and culture medium from uninfected insect cells (as negative baculovirus control). Baculovirus gp64 protein was detected with anti-gp64 antibody (Santa Cruz Biotechnology; sc-65499) (Supplementary **Figure4A**).


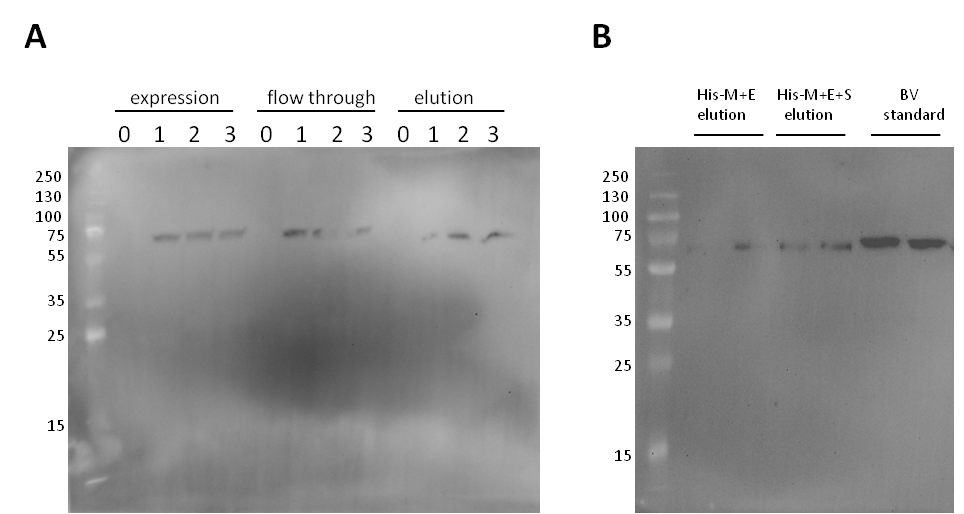


**Supplementary Figure 4. Baculovirus gp64 protein detection in SARS-CoV-2 VLP preparations. A.** Western blot analysis of gp64 protein presence in purification fractions (input, flow through, elution) of samples collected from uninfected insect cells (0 – negative control); cells expressing gus/cat proteins (1 - positive control); cells expressing His-M+E VLPs (2) and His-M+E+S VLPs (3). **B** Western blot with gp64 protein detection used for densitometry analysis and further quantification of baculoviruses present in VLP preparations. Samples of purified VLPs (His-M+E and His-M+E+S elutions) and baculovirus stock of known titer (BV standard) were analyzed in duplicates to increase accuracy of densitometry measurements.

Second, we tried to quantify the number of baculoviruses present in the purified VLP samples. For this we performed another Western blot analysis where baculovirus stock of known titer (previously titrated by plaque assay) were analyzed, together with purified VLP samples. Densitometry analysis (Image Lab 6.0.1, Biorad) of Western blot membrane shown in Suplementary **Figure4B** allowed to calculate the amount of gp64 protein in the purified VLPs samples in relation to gp64 protein in standard baculovirus sample (**Table S3**). Assuming that plaque forming units (pfu, assessed by plaque assay) roughly corresponds to the number bacuroviral particles, we can estimate the amount of baculoviruesto be: 0.15 × 10^8^ /ml and 0.18 × 10^8^ /ml for present in the purified His-M+E VLPs and His-M+E +S VLPs samples, respectively.

**Table S3: Calculation of the amount of baculoviral particles present in purified SARS-CoV-2 VLPs samples.** Densitometry analysis of bands detected with anti-gp64 antibody (Abcam) was performed using Image Lab 6.0.1 software (Biorad). Number of baculoviral particles in purified His-M+E VLPs and His-M+E +S VLPs samples was calculated in relation to standard baculovirus sample (of known titer, assuming that pfu _~_ number of bacuroviral particles in 1 ml).

| Titer of standard baculovirus sample:  (_~_ number of bacuroviral particles in 1 ml) | 1 × 10^8^ pfu/ml |
| --- | --- |
| Band intensity of gp64 protein present in BV standard sample: | 1 |
| Relative band intensity of gp64 protein present in His-M+E elution: | 0.15  (mean value from 2 bands) |
| Relative band intensity of gp64 protein present in His-M+E+S elution: | 0.18  (mean value from 2 bands) |
| Calculated number of baculoviral particles in purified His-M+E VLPs: | **0.15 × 10^8^ /ml** |
| Calculated number of baculoviral particles in purified His-M+E +S VLPs: | **0.18 × 10^8^ /ml** |
